# Cultural Engagement Is Related to Decelerated Physiological Age: Doubly Robust Estimations in a National Cohort Study

**DOI:** 10.1111/nyas.70232

**Published:** 2026-03-17

**Authors:** Daisy Fancourt, Saoirse Finn, Hei Wan Mak, Andrew Steptoe, Mikaela Bloomberg

**Affiliations:** ^1^ Department of Behavioural Science and Health University College London London UK; ^2^ Department of Epidemiology and Public Health University College London London UK

## Abstract

Cultural engagement (e.g., going to live music events and theater performances, museums, galleries and exhibitions, and the cinema) is longitudinally associated in repeated epidemiological studies with age‐related mental and physical health outcomes. However, it is unclear whether it also influences how fast older adults age physiologically—so‐called age acceleration. This study aimed to ascertain whether regular cultural engagement among older adults is related to slower physiological aging using a previously derived physiological age index and a doubly robust estimation approach to account for confounders. Using older adults from the English Longitudinal Study of Ageing (*n* = 4467), we found that cultural engagement was related to lower physiological age cross‐sectionally (average treatment effect −2.17; 95% CI −3.48 to −0.86) and 4 and 8 years later. The effect was seen consistently for all three types of cultural activity explored (cultural performances, museums/exhibitions, and the cinema). These analyses were robust to multiple sensitivity analyses, including considering alternative confounding structures, outliers, and treatment specification. Overall, these findings provide insight into how cultural engagement may be related to processes of aging.

## Graphical abstract

Cultural engagement (including going to cultural performances, museums/exhibitions, and the cinema) is related to age‐related mental and physical health outcomes, but it is unclear if it influences age acceleration. Using older adults from the English Longitudinal Study of Ageing (*n* = 4467), we found cultural engagement was related to lower physiological age cross‐sectionally and 4 and 8 years later.

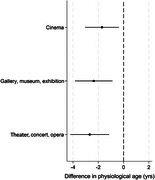

## 1 Introduction

The arts encompass a diverse range of human practices relating to the production or experience of human creativity and imagination, including actively participating in the arts (e.g., dance, music, crafts; hereafter “arts participation”) and attending cultural events (e.g., going to live music events, museums, galleries, theater performances, and the cinema; hereafter “cultural engagement”). Over the past two decades, increasing experimental and epidemiological research has identified diverse mental and physical health benefits that occur as a result of arts participation and cultural engagement, leading to calls for the arts to be formally recognized as a health behavior [1, 2, 3]. Theoretically, such effects may occur due to the “active ingredients” that arts participation and cultural engagement comprise and the mechanisms of action these ingredients activate. From an ingredients perspective, arts and cultural engagement act as a vehicle to well‐known salutogenic behaviors such as social interaction, physical activity, and cognitive stimulation. But they also bring people into contact with other ingredients such as creativity, imagination, multisensory stimulation, and aesthetics that are strongly expressed in the arts [4]. These ingredients synergistically activate diverse psychological, biological, and behavioral mechanisms that are linked to health‐related outcomes [5].

Focusing on cultural engagement specifically among older adults, this behavior has been longitudinally associated in repeated epidemiological studies with subsequent improvements in mental health and wellbeing, as well as reduced risk of developing age‐related disability or impairment, chronic pain, frailty, chronic diseases like coronary heart disease, cognitive decline, and dementia, and even premature mortality [6, 7, 8, 9, 10, 11, 12, 13, 14, 15, 16, 17]. These results have been found independent of measured and unmeasured confounders, using diverse statistical approaches that each account for different types of bias, with some of these findings corroborated by experimental evidence [18, 19]. However, while such evidence suggests that cultural engagement may positively influence age‐related outcomes, what is less clear is whether it also influences how fast older adults age physiologically—so‐called age acceleration.

Over the past decade, there has been increasing interest in the use of biological aging clocks that combine age‐related molecular features and quantify discrepancies between chronological versus biological age, enabling the calculation of whether individuals are experiencing accelerated or decelerated aging. Some aging clocks focus on DNA methylation data, while other clocks have been generated from downstream molecular phenotypes such as proteomic data and metabolomic data, or from broader clinical data [20]. Physiological clocks are an example of a downstream biological clock that combine phenotypic measures of blood‐based biomarkers (e.g., fibrinogen, C‐reactive protein, and glycated hemoglobin) with tests of physiological function (e.g., grip strength, respiratory function, pulse pressure, and blood pressure) that have been found to be valuable in predicting age‐related pathology [21, 22]. These types of clocks are of particular value as they are sometimes more strongly related to disease outcomes of interest than methylation‐based clocks because they look at downstream impacts of cellular aging more proximate to disease outcomes [23]. In previous work, we have generated and validated one such physiological clock and demonstrated that physiological age relative to chronological age predicts many of the same age‐related outcomes that cultural engagement has been related to, including cardiovascular disease, aspects of frailty, cognitive dysfunction, dementia, and mortality [23]. Significantly, in previous work relating deficits in social connections to this physiological clock, the strongest relationship with decelerated physiological age was for social integration, which comprised behaviors such as community group membership, volunteering, and cultural engagement [24]. However, as social integration is a broad index, it remains unclear whether cultural engagement could be one of the active drivers of the observed associations. This warrants further investigation, given the benefits cultural engagement has been reported to have on mental and physical health in older adults.

Consequently, this study aimed to ascertain whether regular cultural engagement among older adults is related to slower physiological aging. Our primary analyses (RQ1) explored associations between an index of cultural engagement and age acceleration/deceleration (calculated as the gap between an individual's physiological age and their chronological age), both cross‐sectionally and 4 and 8 years later. Our secondary analyses (RQ2) explored which type of cultural activity might be driving any potential associations. To improve causal inference, we designated a clear treatment group (i.e., those who engage regularly with cultural activities) and a control group (i.e., those who never engage) and applied a doubly robust estimation approach to account for the nonrandom treatment assignment.

## 2 Methods

### 2.1 Dataset

Data were derived from the English Longitudinal Study of Ageing (ELSA). ELSA is a large‐scale panel study of people aged 50 and over and their partners, living in private households in England. The original sample was drawn from participants from the Health Survey in England (HSE) in 1998, 1999, and 2001 [25]. The first wave of data collection commenced in 2002/2003, and participants have been followed biennially since. We used wave 2 (2004/5), as this wave included a rich battery of cultural and physiological variables of relevance, with longitudinal follow‐up data from nurse visits at wave 4 (2008/9) and wave 6 (2012/13). ELSA receives ethical approval from the NHS Research Ethics Service.

We restricted participants to core ELSA members who had returned the self‐completion questionnaire where most of our main variables of interest were measured and were over the age of 50 at the point of questionnaire completion (*n* = 8129), who had physiological data available (from blood samples and nurse visits) to generate a physiological aging index at wave 2 (*n* = 5572), and who had full data on exposures and confounders (*n* = 5349). Within this sample, we followed a potential outcomes framework approach and defined our exposure (cultural engagement) as a hypothetical intervention [26, 27]. So, we designated our treatment group to be “attending one or more of the three cultural exposures every few months or more” (described further below; *n* = 2480) and a control group to be “never engaging” (*n* = 870). We excluded participants who fell between these two thresholds of engagement (i.e., “less than once a year”) in order to emulate the conditions of a randomized trial more closely. This provided a total analytic sample size of *n* = 3350 (see Appendix S2, Table S1 for sample size across waves and Table S4 for comparison demographics for the full wave 2 self‐completion sample).

### 2.2 Exposures

For cultural engagement, we used data from three questions asking participants’ frequency of visits to (a) the cinema, (b) an art gallery, exhibition, or museum, or (c) the theater, concerts, or opera. For our main outcome index, we considered the treatment group to be “attending one or more of the three cultural exposures every few months or more.” We considered the control group to be those who were completely unengaged (i.e., “never engaging”). We also tested this independently for each activity. Sensitivity analyses tested an alternative threshold of overall cultural engagement.

### 2.3 Outcome

A physiological age acceleration measure (measured in years) was derived using a principal component analysis (PCA) method, an established method to measure physiological aging [28, 29]. It was created using clinical indicators pertaining to the cardiovascular system (pulse pressure and systolic blood pressure), respiratory system (forced vital capacity and forced expiratory volume in 1 s), the inflammatory system (fibrinogen and C‐reactive protein), the circulatory system (hemoglobin concentration), and the musculoskeletal system (grip strength). All measures were taken at wave 2 at nurse visits (blood‐based biomarkers and physiological testing). This index has previously been validated, published, and used in analyses in relation to other exposures [23].

**FIGURE 1:**
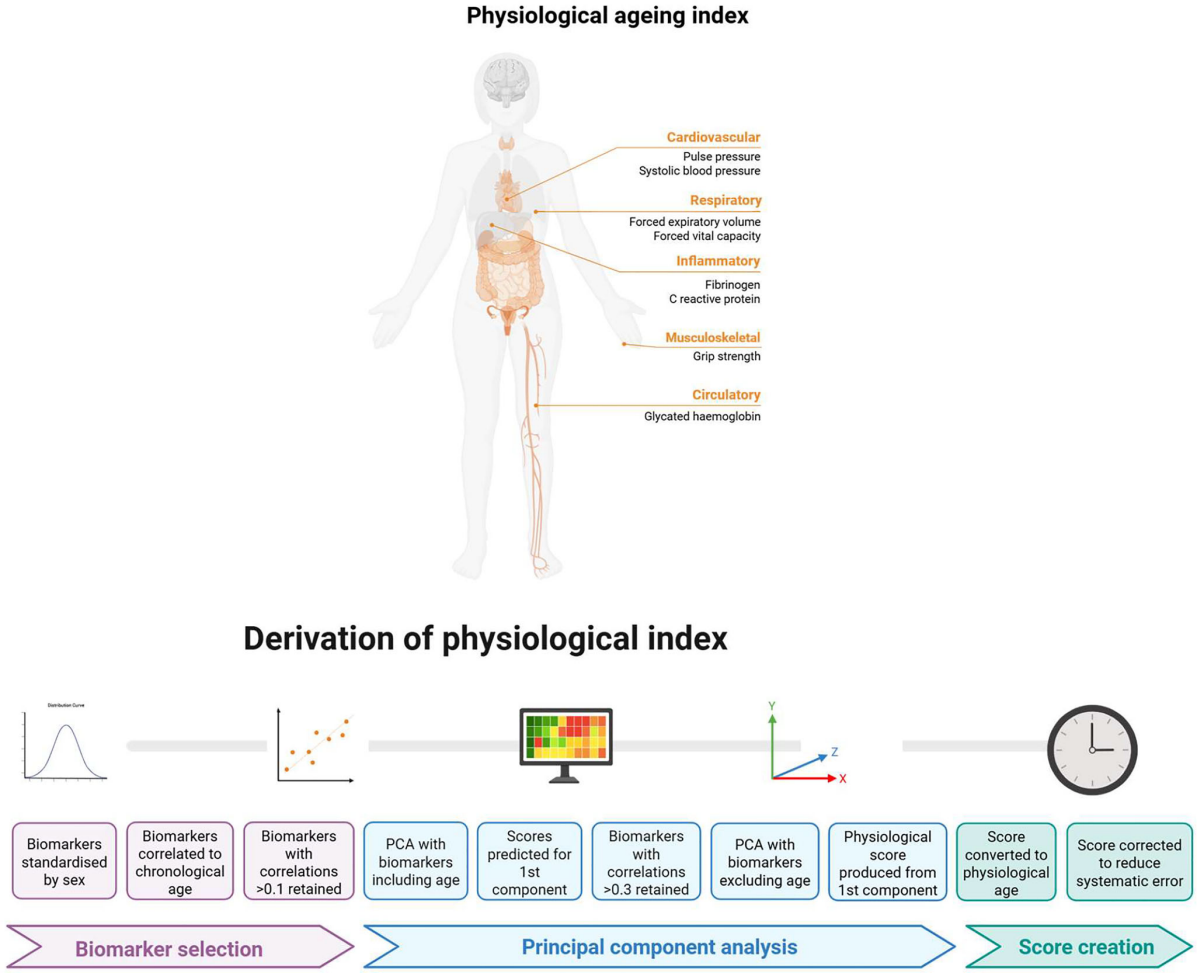
Derivation of physiological aging index.

The full details of physiological age derivation using the PCA method are summarized in Figure 1 and Supplementary Materials (Appendix S1 and Figures S1 and S2 in Appendix S3). To create the physiological age acceleration/deceleration measure, chronological age was subtracted from physiological age; an approach extensively used in previous research [30, 31, 32, 33]. As in previous studies, this means that “accelerated physiological aging” refers to a chronological age higher than a physiological age rather than necessarily implying a longitudinal change. Negative scores indicate decelerated aging (younger physiological age relative to chronological age), and positive scores indicate accelerated aging (older physiological age relative to chronological age). Analyses of this index show its association with incident functional and mobility limitations, memory impairment, and diverse chronic conditions in the ELSA study population, indicating its relevance to aging processes (Table S2).

### 2.4 Confounders

Factors identified as predicting both cultural engagement and physiological age were identified using directed acyclic graphs (DAGs) and included as covariates [34]. Demographic and socioeconomic confounders included sex (male vs. female), ethnicity (white vs. minority ethnic groups), educational attainment (no educational qualifications or qualifications up to age 16, e.g., GCE/O‐levels/national vocational qualification [NVQ] 2; qualifications at age 18 [e.g., A‐levels]; and higher qualification [e.g., NVQ4/NVQ5/degree]), total nonpension wealth (which combines net financial and physical wealth plus net owner‐occupied housing wealth; categorized in quintiles) [35], house ownership (whether individuals owned their property outright vs. with a mortgage/renting/social housing/other), and employment (not working/retired/unemployed vs. working full or part time).

For health and behavioral confounders, we only considered factors that were unlikely to lie on the causal pathway from cultural engagement to aging, which precluded some behaviors known to be influenced by culture. For example, given that cultural engagement provides physical activity through walking incurred in the process of attending events, we only included a minimal adjustment for sedentary behaviors (categorized as not engaging in any kind of mild, moderate, or vigorous activity in a week). We also measured alcohol consumption (more than 5 days a week vs. less), whether participants currently smoked, and whether participants reported currently having a diagnosis of any long‐term conditions, including cancer, arthritis, asthma, osteoporosis, Parkinson's disease, Alzheimer's disease or dementia, lung disease or cardiovascular disease (including high blood pressure, angina, a previous heart attack, heart failure, a heart murmur, an abnormal rhythm, diabetes, a previous stroke, high cholesterol, or other heart trouble). We additionally looked at the number of difficulties in carrying out activities of daily living that participants reported (ADLs; including dressing, bathing, eating, using a toilet, shopping, taking medications, or making telephone calls), self‐reported health (poor, fair, good, very good, excellent), body mass index (BMI) (<25, 25–30, ≥30 kg/m^2^), and depression (using the Centre for Epidemiological Studies Depression scale, ≥3). However, as these are behaviors that have much stronger potential roles as partial mediators of effects, we tested the potential influence that including them had on model results in sensitivity analyses (outlined below).

### 2.5 Statistics

Data were analyzed using doubly robust estimation using the inverse‐probability‐weighted regression adjustment (IPWRA) estimator. This involves building two models to account for the nonrandom treatment assignment: a regression adjustment model for the outcome and a treatment‐assignment model for the exposure, only one of which has to be correctly specified, enhancing the robustness of the analysis [36]. A strength of this approach is that it provides two opportunities to make valid inferences, instead of just one. IPWRA estimators apply weighted regression coefficients to compute averages of treatment‐level predicted outcomes, where the weights are the estimated inverse probabilities of treatment. Following our DAGs, confounders applied to both exposure and outcome were sex, ethnicity, education, wealth, home ownership, employment, physical inactivity, alcohol consumption, smoking, and chronic conditions. Problems with ADLs and self‐reported health were additionally applied to the exposure, and BMI and depression to the outcome.

We estimated the average treatment (ATE) effect in the population, which is the average difference in outcome if everyone in the population experienced the exposure, versus no one in the population [36]. For all analyses, we applied probability weights for the self‐completion questionnaire for wave 2 provided in the data, which account for complex sampling strategies and nonresponse. For RQ1, we looked at overall cultural engagement associations with physiological age cross‐sectionally (at wave 2) and then both 4 (wave 4) and 8 (wave 6) years later. As we used doubly robust estimation, we ran these three analyses separately rather than in mixed models. For RQ2, we repeated the cross‐sectional analyses for each type of cultural activity separately. Sample size was allowed to vary between analyses to maximize the sample.

We also conducted several sensitivity analyses. First, we acknowledge that baseline adjustment of the outcome is controversial as it may lead to downward bias in the presence of residual autocorrelation or overcontrol bias, but equally, leaving it out puts analyses at risk of omitted variable bias or overestimation of the effect of the exposure [37, 38]. As such, our core analyses did not control for baseline physiological age, but a sensitivity analysis did adjust for physiological age 4 years earlier, providing insight into whether cultural engagement relates to *change* in physiological age acceleration over time. Second, depression and BMI could plausibly lie on the causal pathway from cultural engagement to accelerated aging, so they were only included in the outcome confounder model, not the exposure confounder model, in the main analyses. However, a sensitivity analysis additionally accounted for depression and BMI within the exposure model. A third sensitivity analysis excluded anybody whose physiological age was more than 30 years above or below their chronological age to assess the stability of the findings without the influence of outliers. As doubly robust estimations do not allow for polytomous exposures (as it works to emulate a trial design with treated and untreated groups), a fourth sensitivity analysis applied a linear regression model to look at dose−response relationships (never, once or twice a year, every few months, monthly or more). A fifth sensitivity analysis (using doubly robust estimations again) dichotomized the results by age (split at 65 years) to ascertain whether results were consistent across aging. A sixth sensitivity analysis repeated the main analysis using an alternative algorithm to calculate proportional discrepancy in age: age index‐chronological age/chronological age [39]. A final sensitivity analysis considered the stability of results when memory was added to the derivation of the physiological aging index. Since cognition is not just a physiological process, it was not included in the original index. But cognitive decline provides an additional indicator of aging from another organ (i.e., the brain), extensively researched in accumulating literature on brain clocks [40], providing a potentially important additional component to considerations of physiological aging, so we tested to see if it materially affected results.

## 3 Results

### 3.1 Descriptive Statistics

Of the 3350 adults included in the analyses, the average age was 65.4 years (SD 8.8), and 56.1% were female. Those more engaged in cultural activities had multiple advantages in socioeconomics, health, and health behaviors.

**FIGURE 2:**
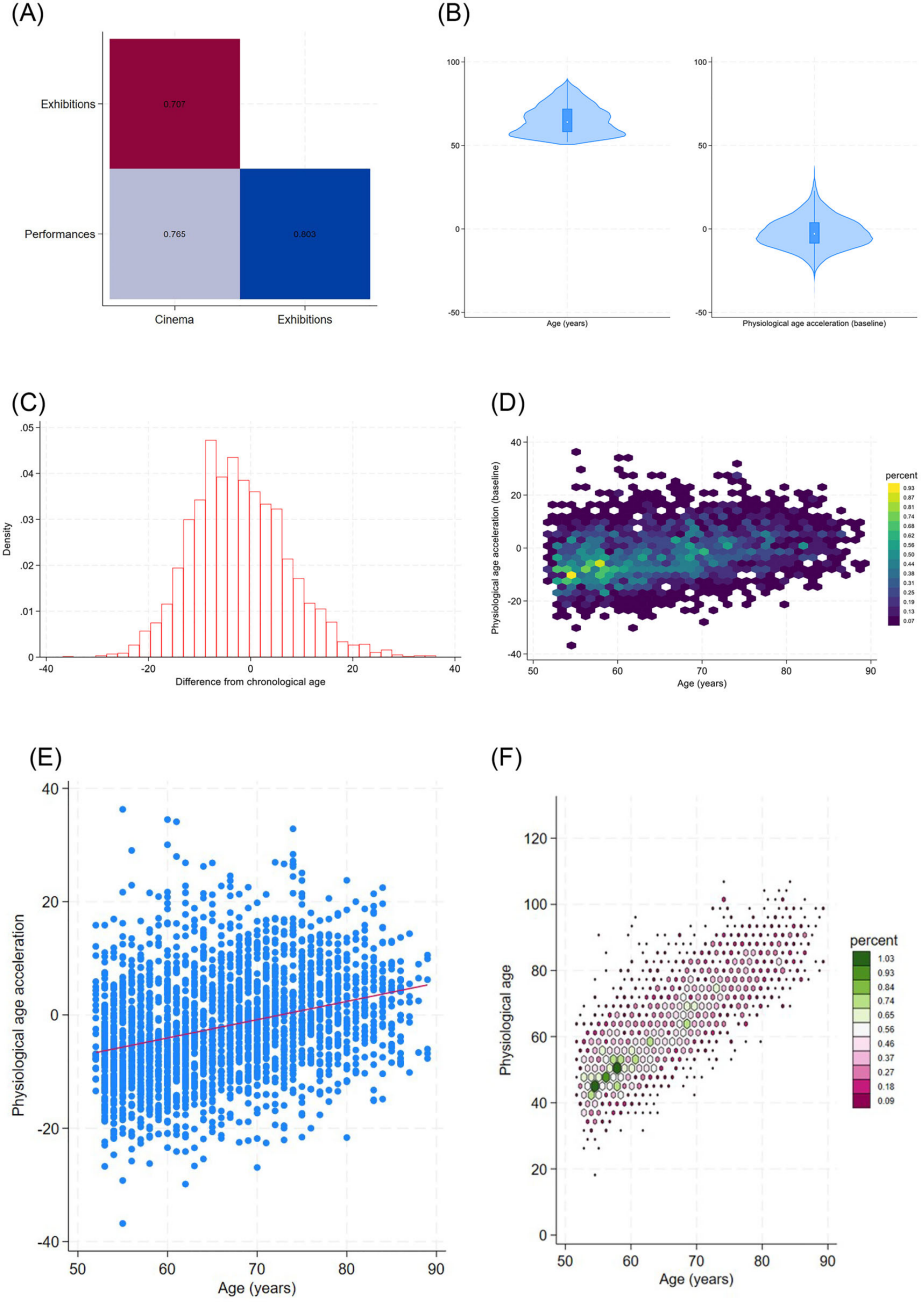
Descriptive figures of exposures and outcomes. (A) Heat plot of correlations between cultural engagement. (B) Violin plots of chronological age and physiological age acceleration. (C) Histogram of distribution of physiological age acceleration. (D) Hexagon heat plot of distribution of physiological age acceleration by chronological age. (E) Scatterplot with linear prediction of chronological age acceleration relative to chronological age. (F) Bivariate histogram using hexagon heat plots of the relative frequency of associations between physiological age and chronological age.

Correlations between the different cultural engagement activities included in the model are shown in Figure 2A (full correlation matrix for all variables shown in Figure S3). The distribution of physiological age is shown in Figure 2C,D. Physiological age was fairly evenly distributed above and below chronological age, with a slight skew toward people being older physiologically than chronologically (62% the same or below, Figure 2B). There was a marked correlation between older chronological age and older physiological age (scatterplots in Figure 2E, F).

### 3.2 RQ1: Overall Cultural Engagement and Physiological Age Acceleration

**FIGURE 3:**
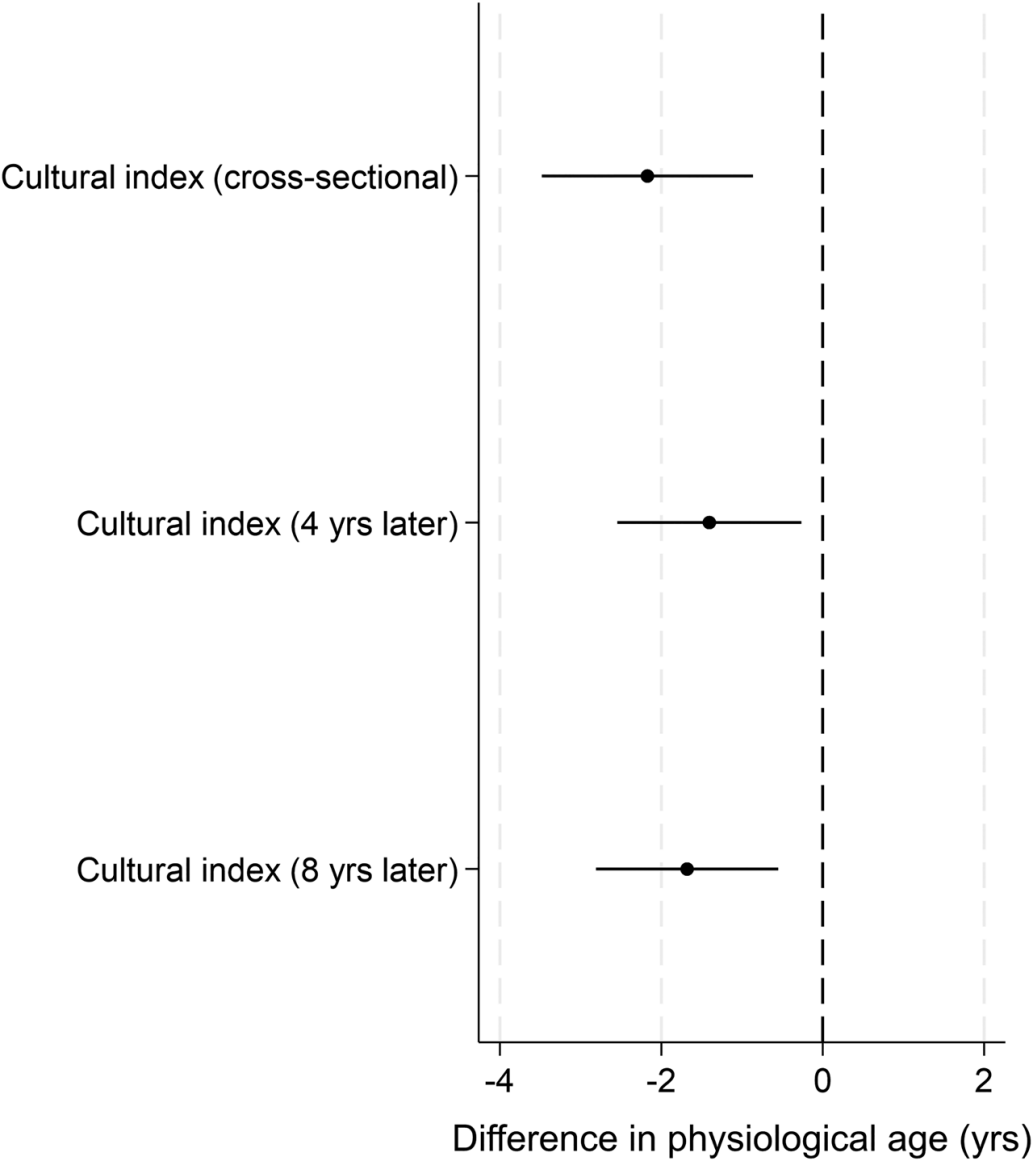
Associations between overall cultural engagement and physiological age acceleration. Dots represent the average treatment effect (ATE) and lines represent 95% confidence intervals (CI).

Cultural engagement every few months or more was associated with decelerated physiological age (i.e., a lower physiological age relative to chronological age) cross‐sectionally, and 4 and 8 years later, even when accounting for confounders within the models (Figure 3 and Table S5). Those who were regularly engaged in cultural activities had a physiological age 2.2 years younger than those who were unengaged (ATE −2.17; 95% CI −3.48 to −0.86). These effects were maintained 4 and 8 years later (physiological age 1.4 and 1.7 years younger than those who were unengaged, respectively), again independent of identified confounders.

### 3.3 RQ2: Specific Cultural Activities and Physiological Age Acceleration

**FIGURE 4:**
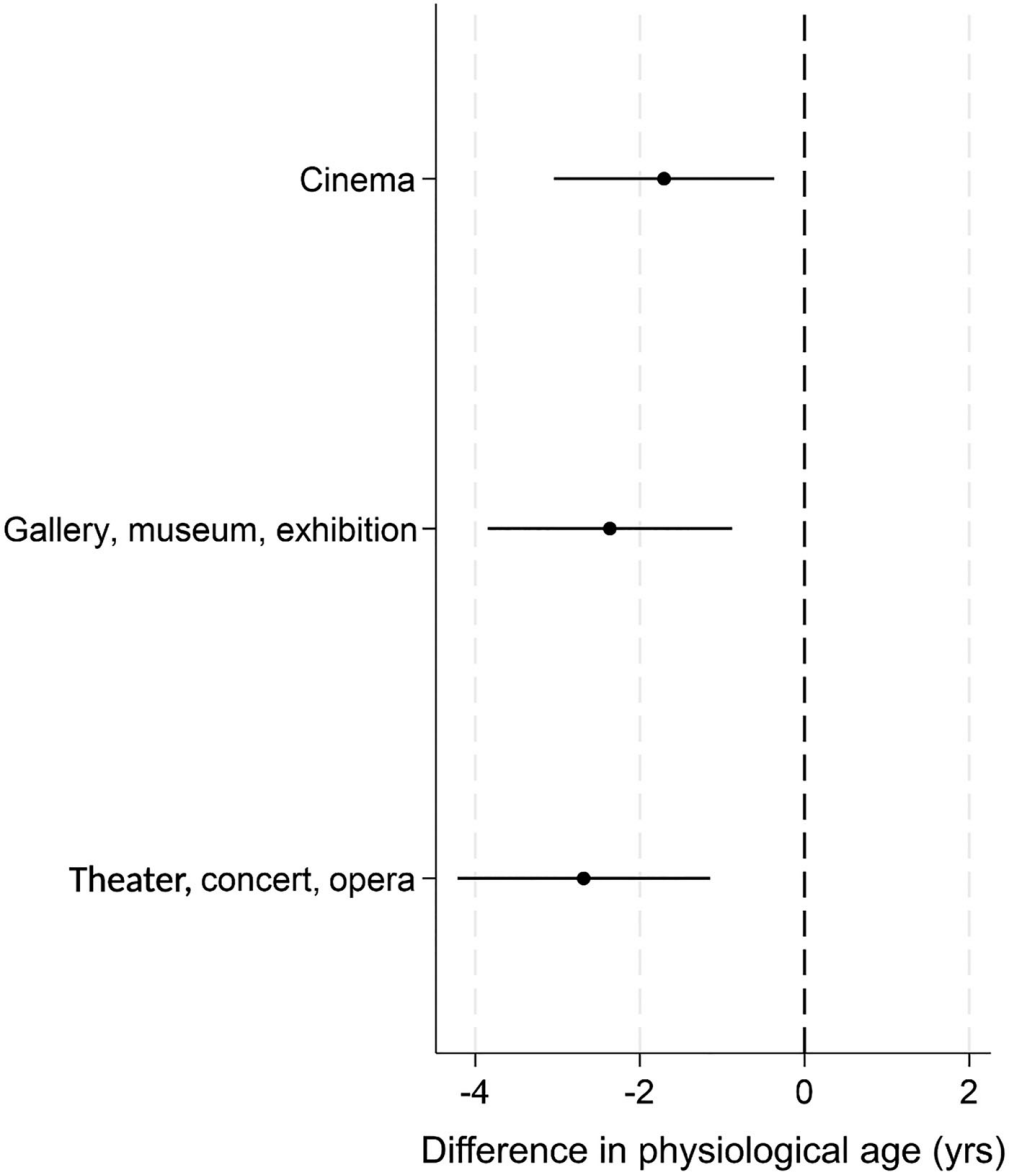
Cross‐sectional associations between specific types of cultural activity and physiological age acceleration. Dots represent the average treatment effect (ATE) and lines represent 95% confidence intervals (CI).

When considering which type of cultural activity contributed most to the model, all three were associated with slower physiological aging. Going to performances showed the largest association with decelerated physiological age, with those who went regularly having a physiological age 2.7 years younger than those who never went. Similarly, those who went regularly to the cinema and exhibitions were 2.4 and 1.7 years younger compared with controls, respectively (Figure 4).

### 3.4 Sensitivity Analyses

Sensitivity analyses showed that associations at longitudinal follow‐up were maintained when controlling for physiological age at baseline, demonstrating that overall cultural engagement was additionally related to a slower pace of physiological aging over the following 4 years of 1.1 years (wave 2–4) and 0.9 years (wave 4–6) (e.g., 4‐year follow‐up adjusted for baseline: ATE −1.10, 95% CI −2.20 to 0.00, *p*<0.05; Figure S4). Adding different confounders into models or excluding outliers also had no material effect (Figures S5 and S6). A linear regression model showed a clear dose−response whereby there was a stronger association the more frequently people engaged compared to people who never engaged (Table S6; *p* for trend <0.001). There appeared to be some attenuation at twice a month or more, but the sample size for this category was very small. When considering the analyses stratified by age, associations were strongest for those aged under 65, where overall cultural engagement was related to physiological age deceleration of 2.7 years (95% CI −5.20 to −0.24) compared to 1.2 years (95% CI −2.24 to −0.20) in those aged over 65 (Figure S7). When applying an alternative computation of proportional discrepancy in age, findings were materially no different (Figure S8). Finally, when including memory as part of the physiological aging index, results were maintained, just slightly stronger (ATE −3.85; 95% CI −5.28 to −2.43; a 37% greater age deceleration than those not engaged; Figure S9).

## 4 Discussion

Overall, cultural engagement was related to lower physiological age cross‐sectionally and 4 and 8 years later, as well as a decelerated pace of aging over the following 4 years. The effect was seen consistently for all three types of cultural activity explored (going to the cinema, galleries, museums and exhibitions, and the theater or live music events). These analyses were robust to multiple sensitivity analyses, including considering alternative confounding structures, outliers, and treatment specification.

The associations between cultural engagement and physiological aging that we found echo previous research that has focused on the individual components of our physiological aging index, finding reductions in cardiovascular risk factors (e.g., heart rate) and favorable inflammatory profiles [17, 41]. However, our findings extend previous research by demonstrating a relationship between cultural engagement and an aggregate aging index that combines these phenotypic markers correlated with age and calculates the discrepancy between the resultant physiological age and an individual's chronological age. As this aggregate index has been independently and longitudinally demonstrated to have predictive potential for diverse pathological age‐related health outcomes, we provide the first data indicating that cultural engagement is related to the speed of physiological aging processes. It is important to note that these findings are likely bidirectional, as for many mechanisms relating cultural engagement with health, with physiological functioning potentially not only influenced by cultural engagement but also affecting one's ability to engage in cultural activities [42, 43]. However, when we used outcome measures from subsequent years, results were maintained, with only modest attenuation of effect sizes within the first 4 years of follow‐up, and very little further attenuation at 8 years of follow‐up. Further, the relationship was similarly maintained in longitudinal analyses that additionally adjusted for baseline physiological age, demonstrating that cultural engagement was not only related to decelerated physiological age but also decelerated pace of physiological aging, strengthening causal inference.

Strengths of our study include the use of data drawn from a large, representative sample of older adults, its use of a multidimensional index of physiological age that has demonstrated associations with diverse aspects of age‐related pathology, and its statistical use of doubly robust estimators, which provide a clear advance on more traditional regression‐based approaches to conditioning on counterfactuals. However, there are several limitations. For example, we were only able to explore cultural engagement, not active participation in the arts, and our measure asked about behaviors in isolation from the contexts in which they occur. As such, the current analyses do not consider whether relationships found are dependent on particular socioecological contexts of cultural engagement. Future analyses are recommended that consider broader types of arts engagement and use experimental methods to elucidate how context influences effects, as well as exploring if results differ among different demographic subsamples. Additionally, we relied on retrospective reporting of cultural engagement rates over the past year, which could be prone to recall bias, especially among older participants [44]. We used a statistical approach that had many strengths in terms of confounder consideration, but did require considering our exposure as a dichotomous variable to enable the creation of a treatment and nontreatment group. This, naturally, has its own limitations, including precluding comprehensive examination of dose−response effects and leading to some attrition (although the sample demographics did not meaningfully change compared to the broader ELSA sample). But it provides an initial step toward future target trial emulation approaches using cultural engagement as an exposure. Additionally, while we included identified confounders and used a statistical method that allows two different model specifications for exposure and outcome (only one of which has to be correct), residual confounding remains possible, so methods such as instrumental variable approaches that better account for unmeasured confounding are also recommended for future work to triangulate with these results and confirm findings. Cross‐sectional analyses may be affected by selection bias, and longitudinal analyses may be affected by attrition bias, if individuals with lower engagement and poorer health are both less likely to survive to participate at baseline and more likely to drop out during follow‐up. However, this would have attenuated observed associations and, therefore, does not contradict the findings. Nonetheless, future longitudinal analyses considering survival within models are encouraged. In relation to our physiological aging index, PCA is a common method for biological age derivation, but it is by no means the only approach and has its own limitations, such as including chronological age within a first component, potentially capturing variance unrelated to aging, and underestimating the hierarchical nature of biological processes. Nonetheless, we selected PCA above alternatives, such as latent variable approaches, because even though these other methods better account for hierarchical dependencies, the summary measures produced are not easily interpretable with respect to chronological age. Further, there is a strong precedent for its use in similar derivation of indices [28, 32, 45, 46, 47, 48, 49, 50, 51]. Future studies are also encouraged to explore how cultural engagement may influence patterns or trajectories of physiological aging, and whether engaging consistently over time is important to effect sizes. An important part of such future work will be able to explore whether temporal variation in confounders affects outcomes, whether effects are bidirectional (as we hypothesize they likely are), and whether feedback effects are evident. Additionally, for future work, while this study focused on an overall index of physiological age, in keeping with other aging research [28, 32, 45, 46, 47, 48, 49, 50, 51], studies are also encouraged to look at organ‐specific aging to ascertain whether cultural engagement affects different biological systems at different rates.

Overall, these findings are important as they provide insight into how cultural engagement may be related to physiological processes of aging. From an intervention design perspective, monthly cultural engagement compared to every few months did not lead to stronger outcomes, suggesting that there may be a minimum amount of cultural engagement necessary, beyond which further engagement does not yield stronger benefits. Further, the strength of the relationship with physiological aging appears stronger among those aged 50–65. These details could help to guide the design and implementation of future experimental studies.

## Author Contributions

M.B. and A.S. derived the physiological aging index. D.F., S.F., and H.W.M. designed the current study. D.F. ran the analyses and drafted the manuscript. All authors critically appraised the manuscript and approved it for submission.

## Conflicts of Interest

The authors have no conflicts of interest to declare.

## Supporting information




**Supplementary Material**: nyas70232‐sup‐0001‐SuppMat.docx
